# Hospital Expenditure.—The Commissariat

**Published:** 1896-08-29

**Authors:** 


### The Institutional Workshop.

### HOSPITAL EXPENDITURE.—THE COMMISSARIAT.

### XXV.—THE COST OF EGGS.

In examining the figures which give the annual cost of this article of diet in various hospitals, it is at first sight matter for surprise that they are no higher. It would appear from the printed diet sheets that this cheap, light, and palatable form of nourishment holds no place in the patients' bill of fare unless ordered as an extra, and when it is borne in mind that, with the exception of milk, it forms the only complete natural food, it is difficult to understand the reason for this omission. The following table, after Landoïs, shows the comparative value of the white and yolk of eggs:—

| | White of egg. | Yolk. |
|---|---|---|
| Water ... ... ... | 84·8 ...... | 51 5 |
| Albuminates ... ... | 12·0 ...... | 15·0 |
| Fat, &c. ... ... ... | 2 0 ...... | 30 0 |
| Mineral matter ... ... | 1·2 ...... | 1·4 |
| Pigment extractives ... | — ...... | 2·1 |

According to Dr. Parkes an egg weighing 2 oz. contains 200 grains of solids, while Pavy* calculates that such an egg would yield 110 grains of nitrogenous substances, 82 grains of fat, and 11 grains of saline matter. Many patients who turn with distaste from their daily portion of meat might welcome nourishment presented in this concentrated form, which furnishes a higher value of food properties at something under half the cost of meat.

Eggs are used in hospitals mainly in the preparation of puddings both for staff and patients, as a breakfast dish for the staff, and (less commonly) beaten up with some stimulant for patients unable to take solids. The extent to which they are used varies greatly in different hospitals. As an instance of the difficulty of arriving at any probable mean of consumption, the following figures may be quoted showing the number of eggs used yearly for staff and patients respectively at Guy's and the Birmingham General:—

| | Yearly average number of eggs consumed per head. Household. | Patients. |
|---|---|---|
| Guy's Hospital ... ... ... | 145 | 196 |
| Birmingham General Infirmary | 182 | 40 |

The average at Birmingham of one each, every other day for the staff, is not nearly reached at Guy's, while on the other hand the patients' average, which exceeds this estimate at Guy's, is no more at Birmingham than one each every nine days.

Certain provincial hospitals state in the annual report the exact amount used during the year of various articles. It is interesting to compare the proportion per head used in these institutions:—

| | Average yearly number of eggs consumed per head by household and patients. |
|---|---|
| Sussex County ... ... ... ... | 251 |
| Belfast Royal ... ... ... ... | 179 |
| Birmingham Queen's ... ... ... | 167 |
| Norfolk and Norwich ... ... ... | 139 |
| Oxford Radcliffe ... ... ... | 136 |
| Halifax ... ... ... ... ... | 74 |
| Leeds General Infirmary ... ... | 43 |

No London hospitals quote amounts used in the report, and it is difficult to estimate the number of eggs on account of the common practice of supplementing the contract supplies with new-laid eggs, at any rate, in the scarce seasons. Making no allowance for new-laid eggs, which do not appear to be used in all hospitals, it seems that the yearly average per head in London hospitals varies from about 117 at the Seamen's and 227 at St. George's, to as much as 431 at St. Mary's, and at the London Hospital, where only the doctors and the household servants are boarded in the general account besides the patients, to 503. It is not clear why so many eggs are required for the patients' use at the latter institution; they do not appear in the diet table except as ingredients of certain puddings, and at the London Nursing Home the average per head does not rise above 215.

It is easy to see how the required quantity of eggs may vary a good deal without any very important distinction in diet. In the matter of "milky" puddings, for instance, everything depends on the kind of farinaceous matter used. Rice, tapioca, sago, and semoulina are far more appetising prepared without eggs; and if any one doubts this let him call in at the London Hospital when the model rice-puddings made in that institution are being portioned out, and he will get a convincing demonstration. Bread-and-butter puddings, on the other hand, maccaroni, corn-flour, and in fact most of the prepared flours, not to mention custard, require eggs in the proportion of at least one to a pint of milk. The difference, then, to the hospital of pudding with egg and pudding without egg, supposing it to form part of the daily bill of fare, amounts to a matter of over 18,000 eggs for each 100 patients in the course of a year, but even so the yearly number consumed by each patient would not amount to more than about 180.

The cost of eggs varies in London hospitals from 8s. per 120 to 10s. 1½d., which embraces a variation of from 12 to 15 a shilling; the most common quotations lie between 8s. 6d. and 9s. 6d. The advantage of a double contract for summer and winter is specially conspicuous in the matter of eggs. In spring and early summer they can be had new laid in most country districts for 16 a shilling, and the actual summer price at Middlesex is 17 a shilling, or 7s. 3d. per 120. In autumn and winter the use of foreign eggs is inevitable, but if the contract is an all-round one, the contractor probably supplies foreign or so-called "fresh" eggs throughout the year, and the hospital entirely misses the advantage to be gained by the plentiful season. In many hospitals better eggs are provided for the use of the staff, and in this one particular the double price would seem to be justifiable. The eggs used by patients are consumed almost solely in the form of puddings, and every housekeeper knows that eggs which are perfectly sound when used for cooking, may turn out very far from palatable when boiled; now a breakfast wrecked by eggs, flavoured with straw, is a very poor preparation for a hard day's work.

The price of eggs in country towns is appreciably higher than in London, doubtless because the supply is drawn from home sources, and what is commonly called the "shop egg" is almost unknown. It is not

* "Food in Health and Disease," Dr. Burney Yeo.

quite clear in all cases whether 100 or 120 are included for the quoted price. The minimum is the summer price at Liverpool Royal Infirmary, 7s. 2d.; the winter price at the same institution rising to 14s. 6d. Nowhere else does the price fall even to 8s., while at Cambridge it rises to 10s. 7½d.; at Hull to 10s. (per 100); at the Birmingham General to 9s. 6d. summer, and 11s. 4¾d. winter (per 100); and at the Sussex County, Brighton, to 12s. 8½d. At Scotch hospitals the prices vary from 10½d. per doz., at Aberdeen to 9s., 9s. 2d., and 9s. 6d. per 120 at Edinburgh and Glasgow. These prices form yet another proof that the economies effected by provincial over London institutions are not aided by any reduction in the price of common necessaries, for nearly all of which the cheapest market is to be found in London.

The average yearly cost per head, including the whole household as well as patients, in certain representative institutions of this article of diet may be studied in the following table. The list of hospitals quoted is smaller than usual, owing to the fact that eggs are sometimes included in the statement of accounts either with butter and cheese or with poultry. Under the uniform system of accounts they are provided with an entry to themselves, and it is possible to check the exact amount expended in this direction.

| | s. | d. | | s. | d. |
|---|---|---|---|---|---|
| Seamen's ... ... | 8 | 0 | Royal Free ... ... | 16 | 0 |
| Charing Cross... ... | 10 | 0 | University ... ... | 17 | 0 |
| London Hospital Nursing Home ... ... | 14 | 0 | Westminster ... ... | 18 | 0 |
| | | | Middlesex ... ... | 20 | 0 |
| Guy's ... ... ... | 15 | 0 | St. Mary's ... ... | 28 | 0 |
| St. George's ... ... | 16 | 0 | London Hospital ... | 34 | 0 |

Outside London the cost shows a remarkable decrease. Starting again with the minimum, it will be seen that the maximum of 12s. at Oxford and Birmingham Queen's is lower than any but two London hospitals.

| | s. | d. | | s. | d. |
|---|---|---|---|---|---|
| York County ... ... | 3 | 0 | Halifax... ... ... | 8 | 0 |
| Newcastle-on-Tyne ... | 4 | 0 | Sussex ... ... ... | 10 | 8 |
| Hull ... ... ... | 7 | 0 | Birmingham Queen's... | 12 | 0 |
| Birmingham General... | 8 | 0 | Oxford Radcliffe ... | 12 | 0 |
| Bristol General ... | 8 | 0 | | | |

In Scotland the cost rises to something more nearly approaching the London prices:—

| | s. | d. |
|---|---|---|
| Edinburgh Royal Infirmary ... ... ... ... | 10 | 0 |
| Aberdeen... ... ... ... ... ... ... | 12 | 0 |
| Glasgow Western ... ... ... ... ... | 19 | 0 |

In the Belfast Royal the average cost is 16s. per head.

As has been already said, everything depends on the extent to which eggs are used in the ordinary dietary, for cooking purposes, as extras for patients, and as part of the usual bill of fare for the staff. The only way to check expenditure in this direction is to go thoroughly, point by point, into the number required under each of these headings, and ascertain whether the yearly total agrees with this calculation. The sums expended are for the most part undeniably moderate, and the highest pitch of expenditure calls for remark solely in view of the fact that eggs form no part of the regular patients' diet.

Under imperfect kitchen supervision it is possible for a large waste to occur even in what may appear at first to be a not very considerable item of expenditure.

